# Selenoproteins in the Human Placenta: How Essential Is Selenium to a Healthy Start to Life?

**DOI:** 10.3390/nu14030628

**Published:** 2022-01-31

**Authors:** Claire Hogan, Anthony V. Perkins

**Affiliations:** School of Pharmacy and Medical Sciences, Griffith University, Gold Coast Campus, Southport, QLD 4215, Australia; claire.hogan@griffithuni.edu.au

**Keywords:** selenium, pregnancy, placenta, selenoproteins

## Abstract

Selenium is an essential trace element required for human health, and selenium deficiency has been associated with many diseases. The daily recommended intake of selenium is 60 µg/day for adults, which increases to 65 µg/day for women when pregnant. Selenium is incorporated into the 21st amino acid, selenocysteine (sec), a critical component of selenoproteins that plays an important role in a variety of biological responses such as antioxidant defence, reactive oxygen species (ROS) signalling, formation of thyroid hormones, DNA synthesis and the unfolded protein response in the endoplasmic reticulum (ER). Although 25 selenoproteins have been identified, the role of many of these is yet to be fully characterised. This review summarises the current evidence demonstrating that selenium is essential for a healthy pregnancy and that poor selenium status leads to gestational disorders. In particular, we focus on the importance of the placental selenoproteome, and the role these proteins may play in a healthy start to life.

## 1. Selenium and Pregnancy

Pregnancy is a condition of enhanced demand for nutrients and heightened oxidative stress. Deficiencies in micronutrients such as selenium hinder antioxidant responses and can expose the placenta to an accumulation of free radicals and oxidative insult. This can cause placental insufficiency and dysfunction, leading to complications in pregnancy such as pre-eclampsia, preterm birth, gestational diabetes (GDM), inter-uterine growth restriction (IUGR) and small for gestational age (SGA) babies [1,2,3]. An increased understanding of placental selenoproteins in various cellular compartments may lead to the potential use of selenium supplementation as a therapeutic intervention to prevent the development of these gestational diseases. It is our hypothesis that mitochondria and endoplasmic reticulum and their resident selenoproteins play a vital role in cellular responses to oxidative stress, maintaining placental cell homeostasis, and may provide an effective therapy in disease prevention and promoting foetal development.

According to the Australian Government (NHMRC), the RDI of selenium is 60 µg/day in women, which increases to 65 µg/day in pregnant women and 75 µg/day during breastfeeding. Similarly, in the US, the recommended daily allowance according to the NIH is 55 µg/day in women, and this increases to 60 µg/day in pregnancy and 70 µg/day during lactation. When considering the daily intake of selenium, this recommended dosage increases to 95 µg/day to maximally express selenoproteins such as plasma glutathione peroxidase [4,5]. However, selenium intake is highly variable between countries and even the regions that lie within, due to soil selenium content and the subsequent variability in content within plant foods [4]. As a result, daily intake of selenium can range from 7 to 900 µg/day across different areas, and this may have a detrimental effect on human health and the results of supplementation trials performed in various regions. Rayman et al. published a comprehensive review of average selenium intakes across the globe and found that eastern European countries and parts of China are low or deficient in selenium intake (7–30 µg/day). In contrast, adequate to marginally adequate intake is found in Australia, New Zealand and Europe (30–90 µg/day), and high selenium intake is found in parts of North America, Venezuela and Japan (100–724 µg/day) [4]. In countries where the intake of selenium is low, below 45 µg/day, a link has been established between selenium concentration and pregnancy disorders, with evidence of increased placental oxidative stress and poor birth outcomes.

### 1.1. Selenium and Pre-Eclampsia

Pre-eclampsia (PE) is a hypertensive disorder of pregnancy, which is the leading cause of maternal mortality and morbidity [6], occurring in 5–7% of pregnancies worldwide and accounting for 70,000 maternal deaths and 0.5 million foetal deaths annually [7]. The aetiology of pre-eclampsia begins with incomplete conversion of the spiral arteries by superficial ingrowth of extravillous cytotrophoblasts. As the spiral arteries establish the maternal supply of oxygenated blood, this incomplete conversion results in poor blood and oxygen flow, increased generation of ROS and evidence of oxidative stress. Due to poor placentation, there is over-production of anti-angiogenic factors, systemic/placental inflammation and generalised endothelial cell dysfunction that produces the characteristic pre-eclamptic symptoms of hypertension, proteinuria and oedema [8].

Many studies have demonstrated that selenium concentrations in women with pre-eclampsia are significantly lower than those women experiencing a normal pregnancy, suggesting that selenium status is associated with the incidence of pre-eclampsia [2,3,9,10,11,12]. A study on the global incidence of pre-eclampsia matched to reported values with selenium intake and plasma/serum selenium concentrations (µg/L) has shown the importance of selenium intake and pre-eclampsia [13]. Data were obtained from 45 countries spanning Europe, Asia, Australasia, Africa, North and South America (6,456,570 births), and demonstrated that there was a highly significant correlation between increasing plasma selenium concentration and reducing pre-eclampsia incidence (Pearson’s r = 0.604 *p* < 0.0001). Serum/plasma selenium levels of >95 µg/L were considered selenium sufficient, and significantly reduced pre-eclampsia incidence was reported for countries above this value (*p* = 0.0007) [13]. This study and others [14] have demonstrated a potential link between the development of pre-eclampsia and serum selenium levels, suggesting that selenium supplementation may be beneficial in reducing oxidative stress in pregnancy and lowering the burden of this disease.

More recently a double-blind placebo-controlled pilot trial randomised 230 primiparous UK pregnant women to either 60 µg/day of selenium or placebo from 12–14 weeks of gestation until delivery [15]. The primary outcome was to determine whether selenium was protective against development of pre-eclampsia. Whole blood selenium concentration, selenoprotein P and anti-angiogenic factors such as soluble fms-like tyrosine kinase-1 (sFlt-1 is linked to the risk of pre-eclampsia) were measured. At 35 weeks’ gestation, significantly higher concentrations of whole blood selenium and plasma selenoprotein P were observed in the selenium treated group than in the placebo. In addition, concentrations of sFlt-1 were significantly lower in the treated group, suggesting an association between selenium intake and the development of pre-eclampsia [15].

High levels of oxidative stress and endoplasmic reticulum stress because of reduced antioxidant capacity in the placenta are characteristic of pre-eclampsia. Selenoprotein activities in pregnancy have been investigated in numerous studies, where the activity of enzymes such as glutathione peroxidases (GPx), thioredoxin reductases (TrxR) and selenoproteins S, or selenoprotein P [16] has demonstrated a reduced antioxidant capacity in pregnancy [4,5,9,12,17], particularly after the first trimester [17]. Pre-eclampsia also results in 15% of preterm births and preterm pre-eclampsia is significantly associated with IUGR [18].

### 1.2. Selenium and Fetal Development

IUGR is defined as a foetus that has failed to reach its growth potential. It affects 10–15% of pregnancies and is the second most frequent cause of perinatal morbidity after pre-maturity [19]. IUGR is used interchangeably with the term SGA infants; however, diagnosis of IUGR is made in utero when the ultrasound estimated foetal weight is less than the 10th percentile, whereas an SGA foetus or infant is smaller than 10% of individuals born at that gestational age [19]. The most common cause of IUGR is placental insufficiency where insufficient oxygen and nutrients pass from the maternal circulation to the placenta—affecting the development of the foetus [19]. Oxidative stress and reduced antioxidant capacity induced by hypoxic conditions, leading to ROS generation and oxidative DNA injury, are considered to play a fundamental role in the occurrence of placental insufficiency [16,19]. Similar to the situation in pre-eclampsia, ischemia-reperfusion injury contributes to oxidative stress and could result in the release of ROS into the maternal circulation, possibly resulting in oxidative DNA damage, which may underlie development of SGA [20].

The relationship between selenium in the aetiology of IUGR/SGA infants has been analysed due to the importance of antioxidant selenoproteins in the development of gestational disorders [21]. Less extensively than PE, research on inadequate selenium intake has highlighted its potential to result in poor foetal growth and development. One study measured the relationship between first trimester levels (10–14 weeks) of micronutrients including selenium in a cohort of 563 women. It was found that lower selenium levels increased the risk of IUGR by 11% (AOR = 0.89; *p* = 0.013) [22]. The same trend was also recorded in another study by Everson et.al., where the odds ratio of IUGR was significantly lower among mothers with higher selenium concentrations OR = 0.27, 95% CI = (0.08, 0.98) [23]. In another study examining maternal selenium concentrations in SGA compared to appropriate for gestational age babies (control), women whose circulating selenium fell in the lowest Q_1_ quartile (≤56.60 µg/L) had approximately three times higher risk of SGA (OR = 3.02, *p* = 0.019) compared to women in the higher quartiles [24]. Overall, this is convincing evidence of an association between low blood selenium content and the prevalence of poor foetal growth, which may be linked to selenium’s direct effect on placental selenoprotein expression.

### 1.3. Selenium and Preterm Birth

Preterm birth complications are a major cause of foetal death worldwide occurring in 5–18% of pregnancies across 184 countries with more than 60% of these preterm births occurring in Africa and South Asia [25]. An infant that is born preterm is defined as a birth of <37 completed weeks of gestation. Preterm infants have a lower rate of survival than term infants, accounting for 1 million infant deaths a year due to morbidities such as temperature instability, respiratory distress, apnoea, hypoglycaemia, seizures, jaundice, kernicterus, feeding difficulties, periventricular leukomalacia and rehospitalizations [26].

As detailed above, selenium has been implicated in pregnancy complications associated with poor placental function such as pre-eclampsia and IUGR, which are also a common cause of preterm birth. There are a variety of studies that measure the levels of selenium in preterm birth which have presented conflicting evidence. Studies measuring the relationships between first trimester levels of selenium in a cohort of Polish women found significantly lower levels of selenium in pregnancy-induced hypertension in women who delivered in <34 weeks compared to >34 weeks [22]. A similar result was found in a cohort of Dutch women, where the serum selenium concentration at 12 weeks’ gestation was significantly lower among women who had a preterm birth than among those who delivered at term (mean 0.96 µmol/L v. 1.02 [SD 0.13] µmol/L; *t* = 2.9, *p* = 0.001) [27]. Furthermore, a study performed in an HIV-infected population of Lagos, Nigeria, found that selenium deficiency was recorded in 20.4% of HIV patients, and women with selenium deficiency were found to have an eight-fold higher risk of preterm delivery [28]. Africa is known to have the highest rates of preterm birth, where nutrition as well as environmental conditions may have an impact on these results. In contrast to these reports, other studies focused on a Chinese cohort of women indicated that low selenium levels were not a predictor of preterm birth with only 21 (5.3%) of participants having a preterm birth, whereas 266 (66.8%) had selenium deficiency (serum selenium < 70 µg/L) [1]. Therefore, understanding how and why selenium may contribute to pregnancy complications associated with poor placental function may also shed further light on its possible association with preterm birth.

### 1.4. Selenium and Gestational Diabetes

GDM is one of the most common diseases that develops in pregnancy, affecting up to 14% of pregnant women [29]. GDM develops in the second or third trimester and is defined as an imbalance in glucose homeostasis as a result of increased insulin resistance [30]. Although there are many management strategies for GDM, including dietary and pharmacological approaches, there is still a need for effective interventions to prevent the development of GDM [31]. Hyperglycaemia is linked to oxidative stress; therefore, individuals who are pregnant with GDM have increased free radical production [30]. Increased levels of oxidative stress are associated with impaired insulin-dependent glucose uptake, elevated apoptosis rate and placental dysfunction, creating a pro-inflammatory state in GDM women, which can lead to further complications for the mother and foetus [30,31,32].

Supplementary antioxidants such as selenium have been widely studied as a therapy to reverse the oxidative status in pregnancies complicated by GDM. There have been multiple studies that have determined that patients with GDM have lower selenium levels and that selenium supplementation improves glucose homeostasis in these patients [31,33,34,35]. A meta-analysis performed by Kong et.al. reviewed 44 studies; 9 of those including 569 patients meeting the criteria found that selenium levels were significantly lower in women with GDM than those without (SMD  = −1.17; 95 % CI: −1.98 to −0.35, *p*  =  0.005) [29]. In a randomised, double-blinded, placebo-controlled clinical trial performed on 70 women with GDM, it was found that 200 µg of selenium supplementation significantly reduced markers of GDM [33]. It was also found that selenium supplementation in these patients compared to placebo resulted in significant reductions in fasting plasma glucose, serum insulin levels, HOMA-insulin resistance, high-sensitivity C-reactive protein (hs-CRP) levels and plasma malondialdehyde levels. Significant increases were then found in quantitative insulin sensitivity check index and glutathione, overall demonstrating that selenium could play a role in reducing oxidative and inflammatory symptoms of GDM [33].

## 2. Placental Selenoproteins

Selenoproteins are defined by an active selenocysteine residue and, to date, 25 have been described in the literature. Of these, many have been identified in the human placenta, where they play essential roles in maintaining cell function and viability (see Table 1). Selenium deficiency during pregnancy can lead to low levels of selenoprotein expression, compromising placental function and leading to pregnancy complications. Whilst previous work by ourselves and others has shown clear associations between selenium status and pregnancy outcomes, to our knowledge, this is the first review to focus on the importance of the placental selenoproteome.

### 2.1. Glutathione Peroxidases (GPx 1–4,6)

**Figure 1:**
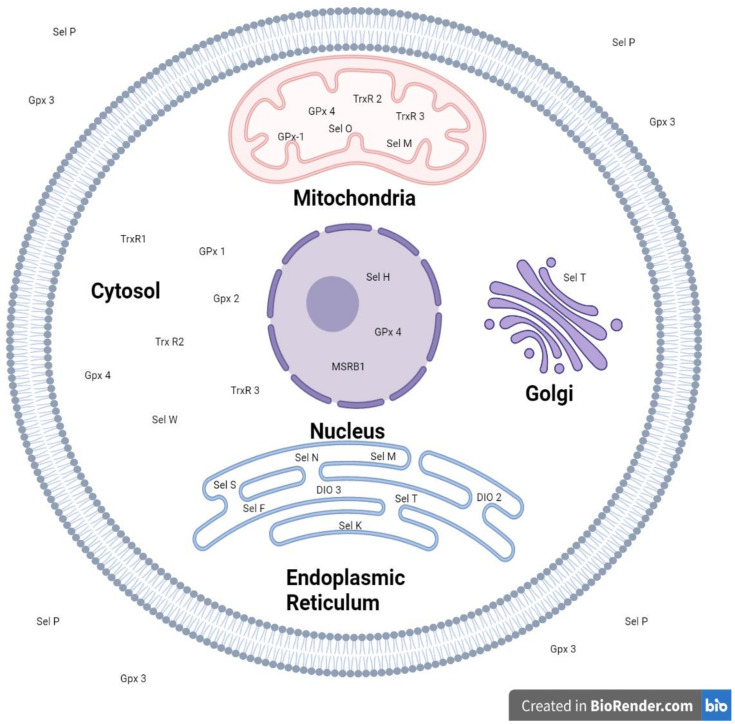
Cellular localisation of selenoproteins in a typical mononuclear eukaryotic cell. GPx1: Glutathione Peroxidase 1; GPx2: Glutathione Peroxidase 2; GPx3: Glutathione Peroxidase 3; GPx4: Glutathione Peroxidase 4; TrxR1: Thioredoxin Re-ductase 1; TrxR2: Thioredoxin Re-ductase 2; TrxR3: Thioredoxin Re-ductase 3; DIO2: Iodothyronine de-iodinase 2; DIO3: Iodothyronine de-iodinase 3; SelF: Selenoprotein F; SelN: Selenoprotein N; SelK: Selenoprotein K; SelM: Selenoprotein M; SelO: Selenoprotein O; SelP: Selenoprotein P; SelT: Selenoprotein T; SelW: Selenoprotein W; SelH: Selenoprotein H; MSRB1: Methionine sulfox-ide reductase B1.

Glutathione peroxidases (GPx) play important roles in oxidative balance and are one of the first and most well characterised groups of selenoproteins. Glutathione peroxidases are part of the glutathione disulphide system that protects cells from oxidative damage by converting hydrogen peroxides into water [58]. There are a total of eight GPx’s identified, and five of these (GPx1, GPx2, GPx3, GPx4 and GPx6) are selenoproteins, as they contain selenocysteine in their active sites [38]. GPx 1–4 have all been identified in the human placenta through RNA and protein analyses [59]. GPx1 is one of the most abundant, is present in all cell types and is found in the cytosol, mitochondria, and in cellular peroxisomal compartments (Figure 1) [35,38]. GPx2 is the main enzyme found in the cytosol of cells lining the gastro-intestinal track, where it is the first line of defence against gut-derived ROS [60]. It has also been found to be expressed in embryonic-derived tissues including the placenta [61]. GPx3 is the extracellular plasma isoenzyme and is a major antioxidant in plasma, which, in combination with SelP, accounts for more than 97% of all plasma selenium, playing a role in selenium transport and homeostasis (Figure 1) [38,62]. Binding of GPx3 is specific and restricted to certain cell populations, which suggests that cells can modify their plasma membranes to provide GPx3 binding sites according to their need for the enzyme [38]. GPx4 is a phospholipid hydroperoxide, which is not only able to reduce H_2_O_2_ but also hydroperoxides in complex lipids [38]. GPx4 has been shown to be vital for embryonic development and male fertility and has also been found to be expressed in placental tissue [5,8]. GPx6 is only found in humans but its function is unknown, despite being identified in the olfactory epithelium and embryonic tissues [38].

Whilst GPx’s have been identified in a variety of tissues, their roles in the placenta have not been fully characterised. Broadly, GPx was identified to be synthesised by the human placenta both extracellular (eGPx) and cellularly (cGPX) and secreted extracellularly into the maternal circulation [63]. In the first trimester placenta, GPx transcripts were localised to cytotrophoblasts, but in full term placentas they were also localised in syncytiotrophoblast cells and stromal cells [64]. Later on, GPx1–4 mRNA and proteins were identified in all layers of the placenta, amnion–chorion, and placental villi in normotensive and pre-eclamptic women at delivery [65]. GPx activity has also previously been identified to be significantly lower in pregnant women in comparison to non-pregnant women, which has long supported the claim that there is a decreased antioxidant capacity in pregnancy [66]. This has led to specific recommendations on increasing daily selenium intakes during pregnancy and breastfeeding.

Multiple studies have identified that GPx expression is compromised in pre-eclamptic pregnancies in comparison to normotensive pregnancies [66,67,68]. One study identified that there were highly significant reductions in overall immunohistochemical staining of Gpx1, Gpx3 and Gpx4 in pre-eclamptic placentas compared to normotensive placentas [58]. It was also found that GPx activity was significantly reduced in placental tissue from pre-eclamptic women compared to normotensive women [58]. Another study also identified that GPx1 gene expression was significantly higher in erythrocytes at term pregnancy, and that this expression was lower in PE compared to controls [66]. Meanwhile GPx4 has been identified to be significantly reduced at term in pre-eclamptic women, regardless of mode of delivery [59,68]. Although GPx activity was found to be reduced in pregnancy and further in pre-eclampsia, it was also identified that the expression of this enzyme could be increased by selenium supplementation in the forms of sodium selenite (NaSe) or selenomethionine (SelMet) in trophoblast cell lines indicating the potential benefit of selenium supplementation in pregnancy [69].

The expression of GPx2 has recently been investigated in developing mouse embryos, where expression of GPx2 mRNA was higher in the extraembryonic tissues than in the embryo [36,60]. In the embryo, expression was spatiotemporally regulated, indicating that GPx2 may be important for organogenesis and foetal development; however, the cell- and tissue-specific functions were not identified [38]. GPx2 has been found to be detected only in placental membranes where levels in amnion–chorion membranes were higher near the umbilical cord than at the periphery in healthy pregnancies, but in PE was found to be homogenously expressed across the placenta [59].

GPx3, the extracellular plasma enzyme, has been demonstrated to play a role in maternal–foetal selenium transfer mechanisms, important in pregnancy and birth, in pre-eclampsia and in the growth of large healthy follicles [36]. Maternal selenoproteins GPx and Selenoprotein P (SelP) were identified in apical vesicles in the epithelial cells in the d-13 visceral yolk sac [70]. In another study with mice, GPx3 mRNA was strongly expressed in decidual cells from days 5 to 8 of pregnancy, and after pregnant mice were treated with a GPx inhibitor for 3 days, the pregnancy rate significantly reduced, which suggested that GPx may play a major role in implantation and placentation [71].

GPx4 is found in placental tissue and is vital for embryonic development and male fertility [16,36]. GPx4 knockout mice are non-viable, and embryos die by gestational day E 8.5, suggesting an important fundamental role of GPx4 in development. Moreover, GPx4 is also related to the activation of the transcription factor essential for inflammatory responses, nuclear factor-kB, where dysfunctional NF-kB expression causes embryonic mortality around gestational day E-15 [36]. More recently, in primary human trophoblasts and mouse pregnancies, it was found that preterm birth was associated with ferroptosis, which is a regulated non-apoptotic form of cell death [72,73]. It was also identified that GPx4 inhibition causes ferroptotic injury in primary human trophoblasts and during mouse pregnancy, which suggests a potential role in the prevention of ferroptosis [72]. GPx4 has been proposed to have an important role in modulating oxidative stress in pregnancy and the development of disease; however, more studies are required to further elucidate these mechanisms.

### 2.2. Thioredoxin Reductases (TrxR 1–3)

Thioredoxin reductases (TrxR) are selenoproteins that are part of a key antioxidant system that regulates oxidative stress. The thioredoxin system is formed by thioredoxin reductase and its associated substrate, the redox active protein thioredoxin, where thioredoxin reductases recycles oxidised thioredoxin at the expense of NADPH [74,75,76]. In turn thioredoxin reduces oxidized peroxiredoxins, peroxidase enzymes with a similar range of activities to the GPx’s. The thioredoxin system is not limited to oxidative stress regulation, playing a variety of roles in cells, including transcription, cell growth control, immune responses, virus infection and in the regulation of cell death [74,77]. There are three thioredoxin reductases that have been identified, including TrxR1, which is cytosolic, TrxR2, which is located in the mitochondria and TrxR3, which is testes specific (Figure 1) [76]. Thioredoxin reductases were originally identified in the placenta, and their localisation and potential roles in oxidative stress in the placenta have been the subject of considerable research. Thioredoxin reductase and thioredoxin activity has been identified in the mitochondria and cytoplasm from subcellular fractions of the human placenta and more specifically were localised histochemically in cytotrophoblasts, decidua, and stromal cells in the stem villi [78]. Importantly, several studies have indicated that the syncytiotrophoblast appears to be devoid of thioredoxin and TrxR immunoreactivity. Significantly less thioredoxin reductase was found in placentae from pre-eclamptic pregnancies when compared to age-matched non-preeclamptic controls [79]. When TrxR activity was measured in trophoblast cell lines such as BeWo and JEG-3, it was found that TrxR activity was responsive to selenium supplementation [69]. At doses of 100 nM of NaSe and 500 nM of SelMet, there were significant increases in TrxR expression, which outlines the potential importance of selenium in the management of placental oxidative stress in pregnancy [69].

### 2.3. Iodothyronine Deiodinases (DIO2 and DIO3)

There are three deiodinases that have been identified across all cell types; however, only DIO2 and DIO3 are expressed in the placenta and may be responsible for the regulation of thyroid hormones that are vital for healthy foetal development [41]. DIO2 is a thyroid hormone-activating enzyme responsible for conversion of inactive thyroxine (T4) to active triiodothyronine (T3) and has been identified in villous cytotrophoblasts during the first trimester of pregnancy [41,42,80]. Meanwhile, DIO3, which has been identified in the first and third trimester in syncytiotrophoblasts, inactivates thyroid hormone by catalysing the inner ring deiodination of T4 to reverse triiodothyronine (rT3) and T3 to 3,3′-diiodothyronine (T2), which are both biologically inactive. A variety of literature has studied the various roles of DIO in the placenta and the importance of thyroid hormone regulation in development [41,42,80].

Thyroid hormones are required for normal foetal development, and the foetus relies on maternal supply until mid-gestation. The exchange of bioactive molecules between maternal and foetal circulations occurs via transport through the plasma membrane and cytoplasm of placental trophoblast cells, and in the first trimester, foetal levels of thyroid hormone can be 100 times less than those of maternal serum [42]. In healthy term placentae, DIO3 activity in the foetal side was significantly higher than that in the maternal side of placenta, suggesting an important role in deactivating thyroid hormones [80]. Other studies have identified that placental DIO3 activity was significantly increased during pregnancy, whereas DIO2 was 200 times lower than DIO3 at all gestational ages and increases in DIO2 were insignificant [81]. These results suggest that DIO2 may not influence thyroid hormone concentration but may play a role in regulating placental T3 generation [41]. Higher levels of DIO3 may suggest a protective role in the foetal tissues ensuring there is no excess in levels of thyroid hormone expression [42]. DIO3 activity in the placenta has also been suggested to contribute to the release of iodide ions into the foetal circulation for foetal TH synthesis [42]. In addition, activities of these enzymes were found to be higher in placental tissues of <28 weeks gestation than at term [41]. Regulation of these thyroid hormones is essential in pregnancy, as mothers with thyroid insufficiency are more prone to pregnancy complications, including spontaneous miscarriage, pre-eclampsia, gestational diabetes, preterm delivery and low birth weight in infants [42].

There is limited evidence on the regulation of selenium in pregnancy in relation to the expression of these DIO enzymes in the human placenta. In some studies, it has been found that mild selenium deficiencies result in partial protection of the thyroid hormone axis and thyroid hormone metabolic pathways. In healthy selenosufficient people and in isolated mild selenium deficiencies, thyroid hormone concentrations were not altered and there was no evidence for impaired activity of these enzymes [82]. It has been suggested that there is a level of protection of the thyroid hormone pathways in these circumstances of mild deficiency, and only chronically reduced selenium availability in combination with genetics has been demonstrated to lead to impaired DIO expression. A recent study by Hofstee et al. examined the effects of a low selenium diet on the expression of a variety of selenoproteins in the placentas of mice. It was found that, in a low selenium diet, increased T4 and T3 concentrations were found, but there was a reduction in DIO2 and DIO3 expression and activity [83]. In GDM patients, DIO3 levels were increased; however, there was a significant decrease in DIO2 expression and activity in the placenta compared to normal mothers [84].

### 2.4. Selenoprotein P

It has been recognized for some time that SelP is produced by the placenta and with GPx3 is released into both the foetal and maternal circulation, playing an important role in transporting selenium [85]. SelP levels increase as gestation proceeds and are at their highest level during the third trimester. Foetal Se accumulation occurs primarily in the foetal liver between the 20th and 40th week of gestation and is highest during the third trimester. Babies born prematurely are seleno-deficient and this introduces metabolic challenges as, in addition, premature babies are often fed parenteral nutrition, have poor intestinal Se absorption when enterally fed, and have immature pathways for Se metabolism [86,87].

There is limited evidence associating abnormal SelP production with gestational disorders. A study by Altinove et al. in 2015 [88] observed no change in circulating Sel P levels in women with GDM; however, another study showed increased levels of SelP associated with the development of preeclampsia and suggested that SelP might be a suitable biomarker for the detection of preeclampsia, albeit in association with other determinants [89].

### 2.5. Selenoprotein H

Selenoprotein H (SelH) has a conserved CXXU thioredoxin-like motif and has been shown to have oxidoreductase activity. It is localised to the Golgi and nucleus where it has role in regulating gene expression in response the redox status, antioxidant defences and oxidative stress. The human placenta expresses SelH but little is known of its function in the placenta and whether changes in placental expression are associated with gestational disorders.

In a recent series of experiments in our laboratory, we supplemented transformed trophoblast cells, BeWO, Jeg and Swan-7, with sodium selenite or selenomethionine in an attempt to protect cells from oxidative stress induced by rotenone and antimycin [69]. The presumption was that increased levels of GPx and ThxR would offer protection and ROS generation would be decreased. This was observed, but we also saw increased mitochondrial function due to increased mitochondrial content in these cells post-selenium supplementation. We probed for mitochondrial biogenesis markers PGC-1α and NRF-1 and saw a correlation between SelH expression and increased expression of PGC-1α and NRF-1 [39,90], an observation that was reported independently by a research group working with neuronal hippocampal cells [91]. This group has now further explored this mechanism and has shown that transfection of SelH into HT22 cells increased the protein levels of nuclear-encoded mitochondrial biogenesis factors, PGC-1α, NRF-1 and TFAM through increased phosphorylation of protein kinase A, Akt/protein kinase B and cyclic adenosine monophosphate response element-binding protein (CREB). Conversely, knock down SelH mRNA level using siRNA reversed these effects [92]. It would appear that SelH is an integral component in regulating mitochondrial biogenesis, and this may be critically important to placental cell health during gestational pathologies that have been shown to be associated with mitochondrial dysfunction and demise.

## 3. Selenoproteins at the Mitochondrial/ Endoplasmic Reticulum Interface

The endoplasmic reticulum is a critical organelle in the secretory pathway and is involved in intracellular protein synthesis and folding, glycosylation, secretion, lipid synthesis and the regulation of intracellular calcium signalling [52,93]. Protein folding is dependent upon the oxidation of disulphide bridges via the ER resident oxidoreductase enzyme ER oxidoreductin 1 (Ero1) and the thioredoxin-like protein disulphide isomerase (PDI) [93]. When proteins cannot be folded correctly due to a heightened oxidative environment due to disruptions in ROS homeostasis [1], these improperly folded proteins are retained in the ER and delivered for proteasomal degradation after retrograde translocation into the cytosol, in a process called ER-associated degradation (ERAD) [94]. ERAD machinery consists of multiprotein complexes that are involved in the recognition, ubiquitination, and retro translocation of misfolded protein from the ER to the cytosol and their subsequent degradation by the ubiquitin/proteasome system [95].

Cellular perturbations such as hypoxia, nutrient deprivation, point mutations in secreted proteins, redox changes, loss of calcium homeostasis can cause ER stress [96]. The unfolded protein response (UPR) is activated by the mammalian ER stress sensors IRE1, PERK and ATF6, causing an adaptive signal transduction pathway [96]. The UPR is activated to recover ER proteostasis as an adaptive response; however, when these adaptive measures are inadequate, the UPR shifts signals to induce terminal UPR and initiate apoptosis [96]. The ER-resident selenoproteins are thought to have oxidoreductase activity and have been implicated in a range of processes including ER stress and the retrograde translocation of misfolded proteins out of the ER, Ca^2+^ homeostasis, inflammation, beta cell function, thyroid hormone synthesis and muscle development [52]. ER-resident selenoproteins have been speculated to have potential therapeutic roles for metabolic disease; however, their roles in regulating ER stress and redox homeostasis in the placenta remain unclear.

As illustrated in Figure 1, the ER resident selenoproteins include SelS, SelK SelM, SelN, SelF, and SelT and have been suggested to play a role in response to ER stress signals, ROS signalling, the unfolded protein response and calcium (Ca^2+^) homeostasis [1]. Despite this, there is still limited information on the roles of these proteins in the placenta and more specifically in trophoblast cells. Selenoprotein S (SEPS1/SelS) is one of the most studied ER resident selenoproteins in the placenta. SelS is an ER membrane protein that participates in the processing and removal of misfolded proteins from the ER to the cytosol, and polymorphisms in the gene have been implicated in the risk of pre-eclampsia and preterm birth outcomes [47,51,97]. Additionally, SelS has been proposed to have a role in mediating inflammatory responses through the protection of the endoplasmic reticulum [47]. A retrospective study on a large Norwegian case–control cohort compared the genotype and allele frequencies of *SEPS1* polymorphism in pre-eclamptic and healthy pregnancy controls [97]. The A allele of the *SEPS1*-105G > A polymorphism was shown to be a significant risk factor for preeclampsia in this population, and genetic variation in *SEPS1 gene* was strongly associated with circulating levels of TNF-α, IL-6, and IL-1β, suggesting a crucial genetic link in influencing these proinflammatory cytokines [47]. There was a significant association of GA or AA genotype (χ^2^ = 7.87, *p* = 0.005) in women with pre-eclampsia, 1.34 times more likely to have the gene in comparison to normal controls, suggesting SEPS1 has a role in protection from pre-eclampsia in that population [97]. Another study investigated *SEPS1* polymorphisms in 569 preterm singleton neonates and 673 term neonates, which found that compared with the GG genotype, -105A positive genotypes (GA + AA genotypes) were associated with significantly increased susceptibility to spontaneous preterm birth (adjusted OR, 1.87; 95% CI, 1.36–2.57; *p* < 0.001) [51]. These studies indicate a potential role of SEPS1 genotypes in inflammation in the placenta and therefore the development of pre-eclampsia or preterm birth; however, more needs to be investigated to understand the mechanism underpinning this.

Other selenoproteins that reside in the ER, such as Sep15, SelK, SelM, SelN and SelT, are less studied when it comes to expression in the placenta. A recent study by Hofstee et al. analysed the expression of many of these ER selenoproteins in pregnant mice who were allocated a low-selenium diet in comparison to a control selenium diet [98]. At embryonic day 18 (E18), maternal and foetal tissues were collected, and the expression of 14 selenoproteins was measured. This study showed that selenium deficiency significantly downregulated selenoprotein expression in maternal tissues and the placenta; however, more specifically, SelN and SelP were significantly reduced. Selenium deficiency did not have an impact on SelF, SelK, SelS, SelM and SelT in the placenta of these mice; however, differences were seen in other tissues. *SelF* was reduced (*p* < 0.05) within the maternal liver, *SelM* was significantly reduced (*p* < 0.05) in all maternal tissues and *SelT* expression was reduced in the heart (*p* < 0.05). Another study measured the selenoprotein mRNA expression in the placenta across the maternal and foetal tissues. It was reported that selenoproteins TrxR1, TrxR2, TrxR3, DIO3, SelH, SelI, SelK, SelN, SelO, SelS, SelV and SelW exhibited the highest expression in the middle section of the placenta, whereas GPx1, DIO2, SelM, SelT, and Sel15, exhibited the highest expression levels in foetal-facing placental tissue and GPx4, SelP, SPS2, and DIO1 exhibited a pattern whereby expression was nearly equal in the foetal-facing and middle section, and lower in the maternal facing portion of the placenta [99]. Further work is required to understand the significance of this localised expression and how this might modulate placenta function.

More specifically, there is limited information on the roles of the other ER-resident selenoproteins. SelK has been demonstrated to participate in oxidation resistance, calcium flux regulation and the ER associated degradation (ERAD) pathway in immune cells; however, not in placenta or trophoblast cells [50]. One study examined the effects of either up- or downregulation of SelK in three trophoblastic cell lines (BeWo, JEG-3 and JAR) [100]. It was found that SelK levels significantly affected β-hCG functions, suggesting that SelK may act as a tumour suppressor in human choriocarcinoma cells by negatively regulating β-hCG expression via ERK, p38 MAPK, and Akt signalling pathways [100]. Similar to SelK, knowledge of SelN is also limited in the placenta; there are data, however, that suggest there is high expression of SelN in foetal tissues and proliferating cells, which suggests a role in early muscle formation [46]. Evidence suggests that SelN serves to regulate RyR-mediated calcium mobilisation required for normal muscle development and differentiation; however, it is uncertain whether the role of SelN is responsible for calcium mobilisation in placental tissues. SelT has been found to be ubiquitously expressed throughout embryonic development and adulthood in rats, and most likely localised to ER through a hydrophobic domain [46]. It, too, as redox activity that might be important in protein folding and maintaining ER function.

## 4. Oxidative Stress in Pregnancy

The placenta is a highly specialised organ of pregnancy that supports the growth and development of the foetus. It does so by connecting the maternal and foetal circulation to allow for the exchange of gases, nutrients, and waste products. The crucial role of micronutrients in the health of an individual in pregnancy is still under investigation, but there is a body of literature describing the critical role of micronutrient intake during all periods of foetal growth and development. Evidence strongly suggests that where there is an excessive increase or decrease in any nutrient, it has a direct relationship on the development of placental co-morbidities. For many years, selenium has been a micronutrient of interest in pregnancy where deficiencies have been directly linked to an increased chance of developing pregnancy complications such as pre-eclampsia, preterm birth and intrauterine growth restriction [101]. A comprehensive understanding of the role of selenium in pregnancy and placental expression of selenoproteins is needed to prevent pre- and post-natal diseases of pregnancy.

Many studies have implicated the role of oxidative stress in pregnancy pathologies. When antioxidant defences are overwhelmed by reactive oxygen and nitrogen species (RONS), this leads to oxidative insults that damage proteins, lipid and nucleic acids structures. Placental development is an intricate process that is highly dependent on oxygen status. During the first trimester, the placental intervillous space is low in oxygen as trophoblast cells plug the tips of uteroplacental spiral arteries, limiting supply of oxygenated maternal blood [102]. At the end of the first trimester, these plugs degrade, followed by a second wave of trophoblast invasion, and an increase in placental oxygen partial pressure triggering oxidative stress in trophoblasts [103]. This sharp rise in oxidative stress in the trophoblast is associated with the onset of maternal blood circulation in the placenta, where ROS act as a signalling molecule that contributes to trophoblast invasion and vascular development in the placenta [18].

## 5. Summary

The placenta expresses low levels of antioxidants in early pregnancy; therefore, pre-mature maternal foetal circulation and widespread oxidative stress overwhelm cellular homeostasis, leading to placental injury. This is the potential cause of spontaneous first trimester abortions, and insufficient placental perfusion and ischemia reperfusion induced oxidative stress are associated with pre-eclampsia and IUGR [102]. Placental oxidative stress is a characteristic of these disorders; therefore, antioxidant status, including selenoprotein expression and activity, has been investigated to determine whether deficiencies in the micronutrient selenium are linked to adverse pregnancy outcomes. A deeper understanding of selenoprotein function at the foetal–maternal interface may path the way for interventions that will decrease oxidative stress, promote placental homeostasis and result in improved foetal development. Ensuring an adequate dietary intake for all pregnant women and using supplementation in cases where seleno-deficiency exists should be a priority in maternal care.

## Figures and Tables

**Table 1:** Placental selenoproteins and their proposed functions.

Selenoprotein	Abbreviation	Function	Subcellular Localisation
Glutathione Peroxidase1	GPx1	Antioxidant protection by preventing the accumulation of hydrogen peroxides. It is the most abundant GPx enzyme. Important roles in female reproductive function, cancer, cardiovascular disease and pre-eclampsia [16,35].	Cytosol, mitochondria
Glutathione Peroxidase 2	GPx2	Antioxidant protection. Main antioxidant enzyme in the GIT [7]. Implicated in the protection of embryos and extraembryonic tissues and expressed in the placenta [16,36].	Cytosol
Glutathione Peroxidase 3	GPx3	Maintenance of cellular redox status, main antioxidant in extracellular fluids/plasma. It is implicated in the implantation in the endometrium, maternal–foetal transfer mechanisms, in common pregnancy and birth, in pre-eclampsia, in preventing oxidative stress induced cell apoptosis during growth of large healthy follicles [16,37].	Extracellular Plasma
Glutathione Peroxidase 4	GPx4	Roles include detoxification of lipid hydroperoxides, antioxidant role in membranes, serves as structural protein in sperm, apoptosis. Present in high concentrations in sperm [16]. Vital for embryonic development and found to be expressed in the placenta [38]. Gpx4 depletion in mice leads to cell death in embryos, testis, brain, heart and photoreceptor cells by lipid peroxidation and oxidative stress Related to activation of NF-kB expression [38].	Cytosol, mitochondria, Nucleus
Glutathione Peroxidase 6	GPx6	Located in the olfactory epithelium and embryonic tissues, but function not known [38]. Only found in humans.	Unknown
Thioredoxin Reductase 1	TrxR1	It is a part of the thioredoxin system and is an antioxidant involved in redox regulation and cell signalling, controls activity of transcription factors, cell proliferation and apoptosis [16]. Has been found to have important roles in embryogenesis [36] and found to be expressed in placental tissue [39].	Cytosol, Nucleus
Thioredoxin Reductase 2	TrxR2	It is a part of the thioredoxin system and is an antioxidant involved in redox regulation and cell signalling [16]. Has been found to have important roles in embryogenesis [36] and found to be expressed in placental tissue [39]	Cytosol
Thioredoxin Reductase 3	TrxR3	It is a part of the thioredoxin system and is an antioxidant involved in redox regulation, cell signalling, disulphide bond formation and sperm maturation [16]. Has been found to have important roles in embryogenesis [36] and found to be expressed in placental tissue [39].	Mitochondria (Testis Specific)
Iodothyronine deiodinase 1	DIO1	Provides a source of plasma T3 by deiodination of T4 in peripheral tissues such as the liver and kidney [40].	Plasma membrane
Iodothyronine deiodinase 2	DIO2	Responsible for the majority of intracellular T3 in tissues such as the brain, pituitary, and brown fat by mediating local deiodination of T4 [40]. Enzymatic activity has been reported in the human placenta in villous cytotrophoblasts in the first trimester [41].	ER membrane
Iodothyronine deiodinase 3	DIO3	Metabolises thyroxine and converts T4 into its. Inactivate thyroid hormone by catalysing the inner-ring deiodination of T4 to rT3 and of T3 to T2 [42]. Found in syncytiotrophoblasts in the first and third trimester of gestation [41].	Plasma membrane
Methionine sulfoxide reductase B1	MSRB1	A repair enzyme that protects proteins from oxidative stress by catalysing the reduction of methionine-R-sulfoxides to methionines. Highly expressed in immune-activated macrophages and contributes to shaping cellular and organismal immune responses [43].	Cytosol
Selenophosphate synthetase 2	SPS2	Catalyses the production of monoselenophosphate (MSP) from selenide and ATP [44].	Cytosol
Selenoprotein F	SELENOF, 15kDA Selenoprotein, Sep15, SelF	Regulates cell stress by enhancing the enzymatic activity of UGGT, and may be involved in glycoprotein folding quality control by rearranging or reducing the disulphide bonds of UGGT-recognised misfolded proteins [45,46]. Sequence homology to protein disulphide isomerases (PDI) [46]. May be involved in apoptosis [47].	ER
Selenoprotein H	SELENOH, SelH, C11orf31	Has a conserved CXXU thioredoxin like motif and has been shown to have oxidoreductase activity. Role in redox status, antioxidant activity and oxidative stress. Expressed in transformed placental cells [16].	Nucleus and Golgi Apparatus
Selenoprotein I	SELENOI, SEPI, SelI, Ethanol-amine-phosphotransferease1, KIAA1724	Catalyses the transfer of phosphoethanolamine from CDP-ethanolamine to diacylglycerol to produce phosphatidylethanolamine which is involved in the formation and maintenance of vesicular membranes, regulation of lipid metabolism and protein folding. Potential role in murine embryogenesis [48,49].	Plasma Membrane and cytosol
Selenoprotein K	SELENOK, SelK	Roles in oxidation resistance, calcium flux regulation and ER-associated degradation (ERAD) [50].	ER and Plasma membrane
Selenoprotein M	SELENOM, SelM	Similar homology to Sep15, may function as a thiol-disulphide oxidoreductase, homologous to PDI’s and may be involved in protein folding [46].	ER lumen
Selenoprotein N	SELENON, SelN, SEPN1	Regulation of RyR-mediated calcium mobilisation required for normal muscle development and differentiation, uncertain if plays a role in calcium mobilisation in other tissues [46].	ER membrane
Selenoprotein O	SELENOO, SelO	Unknown—thought to have redox activity.	Mitochondria
Selenoprotein P	SELENOP, SEPP1, SeP, SelP	Implicated in selenium transport and antioxidant defence. It is a major contributor to plasma selenium and a good indicator of selenium status. Potential implication in pregnancy and pre-eclampsia [16].	Secreted
Selenoprotein S	SELENOS, SEPS1, Sel S, Tanis or VIMP (Valosin-containing protein-interacting membrane protein)	Participates in the processes and removal of misfolded proteins from the ER to the cytosol, protects cells from oxidative damage, regulates inflammation, ER stress induced apoptosis. Additionally, implicated in metabolic and cardiovascular disease, pre-eclampsia and spontaneous preterm birth [16,51,52].	ER and plasma membrane
Selenoprotein T	SELENOT, SelT	Shares sequence homology to thioredoxin-like fold and a conserved cys XX sec motif found in several redox active proteins [52]. Studies in mice indicate a crucial role for this gene in the protection of dopaminergic neurons against oxidative stress in Parkinson’s disease [53] and in the control of glucose homeostasis in pancreatic beta-cells [54].	ER membrane
Selenoprotein V	SELENOV, SelV	Sequence homology to thioredoxin-like fold. Possible role in redox regulation and Testis specific expression in rodents, in situ hybridisation experiments have shown high levels of SELENOV mRNA in seminiferous tubules in mice, but its exact role in spermatogenesis is unclear [55]. Potential in regulating body selenium metabolism in mice [55].	Unknown
Selenoprotein W	SELENOW, SelW, SEPW1	Proposed antioxidant function. Highly expressed in skeletal muscle, heart, and brain, possesses a thioredoxin like fold and conserved CxxU motif, suggesting a redox function. Studies in mice show that this selenoprotein is involved in muscle growth and differentiation and in the protection of neurons from oxidative stress during neuronal development [56,57].	Cytosol

## Data Availability

Not applicable.
